# Cellular polarity pilots breast cancer progression and immunosuppression

**DOI:** 10.1038/s41388-025-03324-0

**Published:** 2025-03-08

**Authors:** Jie Huang, Shufeng Luo, Juan Shen, Maya Lee, Rachel Chen, Shenglin Ma, Lun-Quan Sun, Jian Jian Li

**Affiliations:** 1 Department of Thoracic Oncology, Hangzhou Cancer Hospital, Hangzhou, Zhejiang China; 2 Department of Radiation Oncology, University of California Davis, Sacramento, California USA; 3 Hunan Key Laboratory of Molecular Radiation Oncology, Xiangya Cancer Center, Central South University, China Hunan Changsha,; 4 NCI-designated Comprehensive Cancer Center, University of California Davis School of Medicine, Sacramento, California USA

**Keywords:** Cancer microenvironment, Prognostic markers

## Abstract

Disrupted cellular polarity (DCP) is a hallmark of solid cancer, the malignant disease of epithelial tissues, which occupies ~90% of all human cancers. DCP has been identified to affect not only the cancer cell’s aggressive behavior but also the migration and infiltration of immune cells, although the precise mechanism of DCP-affected tumor-immune cell interaction remains unclear. This review discusses immunosuppressive tumor microenvironments (TME) caused by DCP-driven tumor cell proliferation with DCP-impaired immune cell functions. We will revisit the fundamental roles of cell polarity (CP) proteins in sustaining mammary luminal homeostasis, epithelial transformation, and breast cancer progression. Then, the current data on CP involvement in immune cell activation, maturation, migration, and tumor infiltration are evaluated. The CP status on the immune effector cells and their targeted tumor cells are highlighted in tumor immune regulation, including the antigen presentation and the formation of immune synapses (IS). CP-regulated antigen presentation and delivery and the formation of IS between the immune cells, especially between the immune effectors and tumor cells, will be addressed. Alterations of CP on the tumor cells, infiltrated immune effector cells, or both are discussed with these aspects. We conclude that CP-mediated tumor aggressiveness coupled with DCP-impaired immune cell disability may decide the degree of immunosuppressive status and responsiveness to immune checkpoint blockade (ICB). Further elucidating the dynamics of CP- or DCP-mediated immune regulation in TME will provide more critical insights into tumor-immune cell dynamics, which is required to invent more effective approaches for cancer immunotherapy.

## Background

The mammary gland undergoes a lifetime dynamic remodeling to meet developmental and productive demands by maintaining the gland’s luminal homeostasis, achieved by an orchestrated function of cell polarity (CP) proteins. CP proteins participate in the signaling transduction within the cell and play a key role in cell-cell communications by geographical localization of signaling components and cellular organelles to coordinate with the organ’s physiological functions. Alongside the observed rise in early-onset cancers, the coming decades will witness an estimated 20–50% cancer incidence, including breast cancer (BC), among the >95% malignant diseases that occur in the epithelium [1–6]. The mammary gland is an organ subject to dynamic remodeling throughout its lifetime [7], and disruption of CP in mammary glands is a hallmark of BC with mislocalization and interaction of CP proteins with varied receptors, including HER2 (human epidermal growth factor receptor 2) [8–10], leading to aggressive behavior [8–10]. Breast pathogenesis is accompanied by disruption of cell-cell junctions, repopulation of cancer stem cells (CSCs), and metastatic spread [11–13]. Several findings demonstrate the critical role of CP in regulating immune cell migration and infiltration, which are closely related to immune regulation in the tumor microenvironment (TME) and the efficacy of cancer immunotherapy using immune checkpoint blockade (ICB). In addition to CP-mediated neoantigen presentation on tumor cells and CP protein-regulated immune cell migration and tumor infiltration, CP-regulated antigen presentation, immunological synapse (IS) formation, and effector functions [14]. This review will deliberate the potential interaction between CP-regulation on tumor and immune cells, which may coordinatively cause the immunosuppressive BC microenvironment. We will discuss CP-regulated neoantigen expression cooperated with CP-related antigen presentation in immune cells and CP-guided migration and tumor infiltration of immune effector cells. CP-regulated tumor-immune cell interaction may decide the immune synapse (IS) formation, which is required for an array of essential immunological interactions, including efficacious cancer control by ICB.

## CP proteins for breast gland luminal homeostasis

**Fig. 1:**
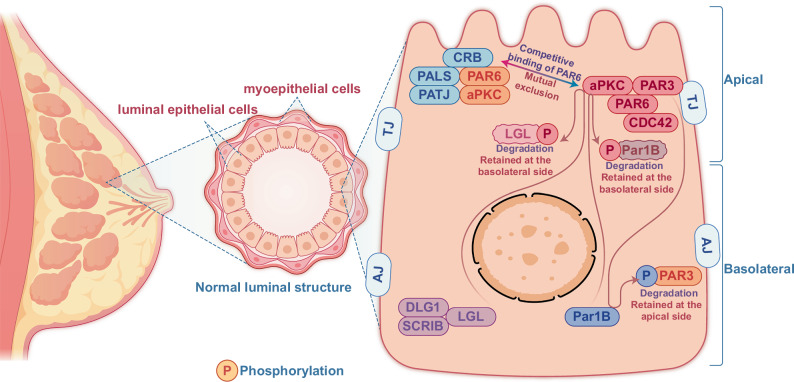
CP proteins maintain breast luminal homeostasis. The mammary ducts of normal breasts are luminal structures composed of an inner epithelial layer and an outer myoepithelial layer. A cluster of CP proteins maintains the luminal structural homeostasis via coordinative activation and localization of different CP proteins for tight interaction among the neighboring epithelial and myoepithelial cells. The CP of the luminal structure is maintained by three major CP components, including Crb and PAR complexes localized to the apical side. In contrast, the SCRIB complex is usually restricted to the basolateral side of the duct. PAR1 phosphorylates PAR3 to prevent it from binding to membrane phospholipids. The pseudo-basal region of aPKC binds to the cell membrane by interacting with PAR6. The apical aPKC also phosphorylates LGL2 and PAR1b to prevent their localization to the apical side.

The mammary duct comprises an inner luminal epithelial layer and an outer (basal) myoepithelial layer. The luminal epithelium’s apical surface faces the lumen, while the basal surface is in contact with the basement membrane and myoepithelial cells. The lateral surfaces are connected to neighboring epithelial cells. CP also referred to as apical-basolateral CP, is primarily regulated by the Crumbs (Crb) and PAR (partitioning defective) complexes located on the apical side and the Scribble (SCRIB) complex, a multidomain CP protein and member of the PDZ (PSD-95, discs large, ZO-1) protein family, on the basolateral side. CP maintains the epithelial barriers to establish intercellular contacts through adherent junction (AJ) and tight junction (TJ) [15]. Crb complex consists of the transmembrane protein CRB3 (crumbs homolog 3) that is connected with PALS1 (protein associated with Lin-7 1) and PATJ (PALS1-associated tight junction) factors [16]. The PAR complex includes PAR3 (partitioning defective 3), CDC42 (cell division cycle 42), PAR6 (partitioning defective 6), and aPKC (atypical protein kinase C) [17]. The SCRIB polarity complex consists of three proteins–SCRIB, LGL (lethal giant larvae), and DLG (discs large) [18]. These three significant complexes exclude each other for sustaining the polarizations of epithelial cells (Fig. 1). The Crumbs complex binds to the PAR complex by competitive binding of PAR6 with PAR3, facilitating the localization of complexes either at the plasma membrane or at TJs by binding to JAMA (membrane phospholipids or junctional adhesion molecule A) [19]. The basolateral PAR1 (partitioning defective 1) and homolog PAR1B (partitioning defective 1b) can phosphorylate PAR3 to localize and maintain PAR3 at the apical side [20]. In contrast, the pseudo-basal region of aPKC binds to the cell membrane with PAR6 interaction. PAR6 binding inhibits the kinase activity of aPKC, which is reversed by the interaction of the apical polarity protein CRB3 (crumbs homolog 3) with PAR6 [21]. In addition, aPKC allows the retention of LLGL2 (lethal giant larvae homolog 2) and PAR1B at the basolateral side by phosphorylating these proteins [22–24]. CRB3 (CRB3a and CRB3b) can promote TJ formation and function [25], and mutations in the PDZ domains of CRB3 attenuate TJ formation [26], which functions as DLG1 (discs large homolog 1) in TJ of mammary epithelial cells [27]. Furthermore, the FERM (band 4.1, ezrin, radixin, moesin) Protein Yurt, predominantly a basolateral protein but is recruited by Crb to apical membranes late during epithelial development, is shown to directly bind to and negatively regulate the crb complex that controls epithelial polarity and apical membrane size [28–30]. However, among the significant proteins for CP regulation, SCRIB (scribbled homolog) localization to the basolateral side specifically prerequisites the E-cadherin controlled cell-cell adhesion [31]. Thus, SCRIB, Crb, and PAR complexes are involved explicitly in sustaining a specific CP pattern via mutually excluding each other to maintain apical basolateral polarity and promote TJ formation. TJ interconnections are sparse in non-lactating mammary glands, whereas TJs in lactating glands are tightly interconnected, preventing fluid leakage [32–34]. Environmental modulators, such as stress and inflammation, can alter TJ and CP, leading to BC risk [35]. The dynamic alterations of luminal structures in breast epithelial transformation have profound immunological implications that affect immune surveillance, tumor progression, and treatment responses. Understanding these interactions may develop better strategies for immune and targeted therapies in BC management.

## Disorganized CP in breast cancer progression

In a study with a cohort of 432 invasive BC cases, loss of apical basolateral polarity was demonstrated in all cancer cells by immunofluorescence staining [36]. CP loss is accompanied by structural luminal collapse with the tumor progression [37]. Decreased CP gene expression is linked with cancer initiation and progression. Disrupted SCRIB is first identified in mammary epithelial transformation and tumor generation [38]. Suppression of SCRIB delays acini development, reduces apoptosis, enhances dysplasia and MAPK (mitogen-activated protein kinase) pathway, and synergizes HRAS (Harvey rat sarcoma viral oncogene homolog)-induced breast epithelial tumorigenesis [38, 39]. PAR3 is reduced in BC tissues and PAR3 knockout mice are more sensitive to HRAS-induced mammary tumors [40]. Consistent with this finding, BC cells with PAR3 deficiency demonstrate mislocalization and activation of aPKC with MMP9 (matrix metallopeptidase 9) activation to digest the extracellular matrix for tumor invasion and metastasis [41]. LKB1 (Liver kinase B1) required for PAR complex is drastically lowered in about 1/3 of sporadic breast tumors. Inhibition of LKB1 in MCF-10A cells boosts acinar hyperplasia and oncogenic behavior [42]. Similarly, CRB3 is reduced in BC cells, and CRB3 knockdown in MCF-10A cells raises cell stemness and invasive capacity [43]. LLGL1 (lethal giant larvae homolog 1), a component of the SCRIB, is reduced in BC [44], and blocking LLGL1 (and also LLGL2) promotes proliferation and migration in epithelial cells [45]. In contrast, about one-third of breast tumors show CP gene amplification, including SCRIB (14.7%), LLGL2 (7%), PRKCI (protein kinase C, iota) (5%), PARD6B (partitioning defective 6 homolog beta) (4.9%), DLG1 (3.92%), and DLG2 (discs large homolog 2) (3.92%) [36]. ER (estrogen receptor) negative tumors with high SCRIB and E-cadherin enhance lymph node metastases with poorer prognosis [46]. SCRIB-associated tumor promotion is related to the PIK3CA (phosphatidylinositol-4,5-bisphosphate 3-kinase catalytic subunit alpha) pathway [47] such as aPKC that is activated in 80% of 110 breast cancer patients particularly in invasive tumors [48]. Overexpression of aPKC in polarized epithelial cells deregulates the Hippo/Yap1 pathway leading to malignant transformation with CP loss [49]. PARD6B, an isoform of PAR6, is also elevated in breast cancers in which PAR6 binding to aPKC and CDC42 activates the MEK (mitogen-activated protein kinase kinase)/ERK (extracellular signal-regulated kinase) pathway raising cell proliferation and acinar hyperplasia [50]. Such CP-mediated aggressive phenotype can be blocked by PAR6 inhibition [51–53]. The small GTPase Rap1 organizing acinar CP and lumen formation is increased in the malignant transformed breast HMT-3522 T4-2 cells, and dominant-negative Rap1 reverses the acinar structures and CP [54]. However, in addition to altered CP gene expression, CP protein mislocalization plays a key role in cancer progression. SCRIB mislocalization is shown in breast epithelial carcinogenesis and tumor progression [47, 55]. Shifting of the apical CDC42 to the basolateral side with phosphorylated aPKCζ cytosolic accumulation and nuclear accumulation CDC42 is found to drive aggressive proliferation of ER positive low-grade breast cancer [56].

## CP patterns driving cancer cell migration and metastasis

**Fig. 2:**
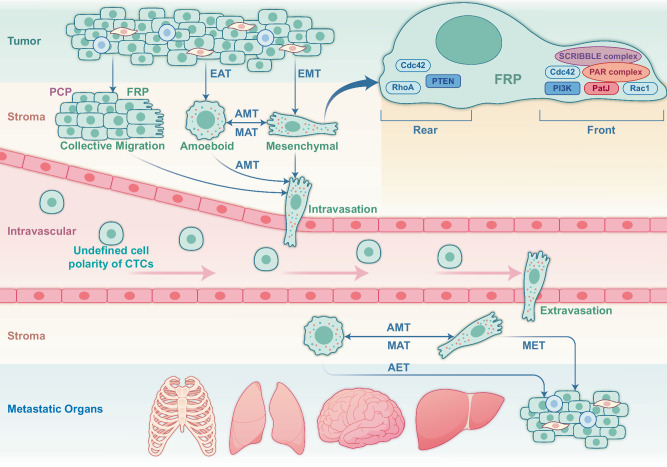
CP in BC metastasis. To initiate metastasis, BC cells need to undergo epithelial-mesenchymal transition (EMT) or epithelial-ameboid transition (EAT) in the status of singular cells or migrate, which is required for penetrating the mesenchyme and enter the circulation in a mesenchymal-like state. Once in circulation, the cancer cells maintain a specific CP pattern to facilitate penetration into the vasculature in a mesenchymal-like status once the tumor cells colonize the metastatic site. Depending on the characteristics of the mesenchyme, the circulating tumor cells (CTCs) may adopt an ameboid or mesenchymal-like state to cross the mesenchyme to colonize the appropriate site as mesenchymal-epithelial transitioned cells, which further alters the CP. The localization of CP proteins may play a vital role in tumor cell migration by sustaining an apical-basolateral polarity. The dynamics of CP in CTCs prepared for local metastasis are to be further elucidated.

CP alternation is tightly linked with all BC metastatic steps encompassing pre-metastatic TME formation, tumor cell migration and infiltration of adjacent tissues, intravasation, survival in the circulation, extravasation, and colonization and proliferation at the metastatic foci (Fig. 2). Epithelial-mesenchymal transition (EMT) is required by the metastatic cells to migrate either as mesenchymal or ameboid cells [57]. Cells with greater contractility tend to choose the ameboid phenotype, whereas those with stronger intercellular adhesion prefer to migrate collectively [58]. In the latter case, DDR1 (discoidin domain receptor tyrosine kinase 1) binds the PAR3/6 complex to localize RhoE to cell-cell junctions, antagonizing actomyosin contractility and promoting collective migration [59]. The collective migration depends not on intercellular adhesion but on cellular front-rear polarity (FRP) [60]. As a dominant feature of migrating cancer cells, FRP organizes the cell machinery along the front-rear axis with a protruding leading edge (front) and a retracting trailing edge (rear), allowing efficient cell motility through a stiff extracellular matrix. Gradient chemokines, growth factors, or other ligands at the front side can facilitate the cells by recruiting PIK3CA kinase and the small GTPase proteins such as Rac and CDC42 to the cell front, thereby promoting adhesion formation with downstream multiple signaling pathways such as PIK3CA/AKT/GSK (glycogen synthase kinase) signaling pathways [61]. In addition, PTEN (phosphatase and tensin homolog) and SHIP (SH2-containing inositol 5’-phosphatase), two phosphatases that convert PIP3 (phosphatidylinositol 3,4,5-trisphosphate) to PIP2 (phosphatidylinositol 4,5-bisphosphate), are retained at the cell rear for reorganizing microtubule-centered reorientation and adhesion disassembly [61]. Recently, FilGAP, a GTPase-activating protein for Rac, is shown to control the FRP and enhance the migration of BC cells in collagen matrices [62]. Planar cell polarity (PCP) is a coordinated alignment of CP orthogonal to the apical-basolateral polarity [63], which facilitates cancer cell migration and metastasis via the Wnt/PCP pathway [64, 65]. The core PCP proteins, Wnt5a, Wnt11, VANGL1/2 (the vertebrate homologs of Drosophila Vang), and PRICKLE1 (prickle planar cell polarity protein 1), are identified to be involved in BC migration [66]. However, whether or not a specific CP pattern is required to drive cell migration metastasis remains unelucidated.

HER2, an oncogenic receptor, frequently amplifies in the HER2^+^ subtype driving aggressive growth [67], is tightly related to CP-associated aggressiveness. HER2 status changes dynamically during treatment, with 66% of patients showing concordant HER2 status between primary and metastatic lesions, and a 9.7% conversion rate from HER2-negative to HER2-positive in metastatic tumors [68]. In invasive micropapillary breast carcinoma, HER2 predominantly localizes to the basolateral membrane [69]. Similarly, in high-grade gastric/gastroesophageal junction adenocarcinoma, HER2 is initially polarized by TJs. However, as the tumor becomes more aggressive, the loss of glandular differentiation and disruption of TJs leads to CP loss [70]. PAR3 downregulation in HER2 + BC activates RAC1 (Ras-related C3 botulinum toxin substrate 1), reducing E-cadherin stability, loosening intercellular junctions, and promoting metastasis [71]. CDC42 can also activate the interactions between cancer cells, endothelial cells, and the extracellular matrix [72]. Inhibition of CDC42 reduces BC metastasis including HER2-mediated cell migration and invasion [72–74] via suppression of ERK5 phosphorylation [75].

## CP patterns driving breast cancer therapy resistance

Serum HER2 protein levels predict HER2 phenotypic transitions, observed in radiation-treated HER2-negative BC where HER2+ breast cancer stem cells (BCSC) emerge [76]. Aromatase inhibitor-resistant BC shows a high HER2^+^ BCSC population [77]. Concurrently, loss of the apical-basolateral CP raises tumor resistance to trastuzumab by enhancing MUC1-C (carboxy-terminal transmembrane subunit of mucin 1) interaction with mislocalized HER2 [78]. The plasticity of BC cells under different treatments appears linked to CP protein dynamics, possibly through HER2-induced CSC repopulation [79]. It is further evidence that ERBIN and PICK1 bind to HER2 via the PDZ domain, restricting HER2 to the basolateral membrane of epithelial cells. Mutations in the ERBIN-binding site cause HER2 mislocalization [80, 81]. Lin-7, another CP protein, supports HER2 retention at the basolateral surface [82]. Together, CP-guided HER2 geographic relocation may provide an alternative approach to enhance anti-HER2 sensitivity or eliminate HER2-expressing BCSCs.

It has been shown that inhibition of aPKC enhances the anti-BC activity of the tumor necrosis factor α (TNF-α) [83], tamoxifen [84] and 5-fluorouracil or adoptive immunotherapy [85]. SCRIB is significantly downregulated in tamoxifen and paclitaxel-resistant BC cells [86]. Either increasing in the cytosolic localization of SCRIB or its depletion shuts down the Hippo signaling pathway leading to an increase in BC stemness [87]. DLG5 (discs large homolog 5), which is transcriptionally regulated by progesterone [88], is expressed at a significantly low levels in tamoxifen-resistant BC cells. DLG5 depletion reduces sensitivity to tamoxifen while increasing the proportion of CD44^+^/CD24^-^ BC stem cells, whereas DLG5 overexpression has the opposite effect [89].

CP proteins are capable of transporting intracellular proteins, lipids, and other accessory biomolecules to function in the organellar polarity distribution [90], which can be modulated by drug endocytosis and efflux. Chen et al. found that basal-like BC cells utilize CDC42 to inhibit Casitas B-lineage lymphoma (c-Cbl)-mediated ubiquitinoylation of target proteins to be degraded in the Redox/Fyn/c-Cbl (RFC) pathway and inhibition of CDC42 restores the RFC pathway function and increases tamoxifen sensitivity in basal-like BC cells [91]. Estradiol induction increases the IC_50_ of adriamycin against MCF-7 cells and decreases the intracellular accumulation of the drug, accompanied by an increase in the CDC42 protein levels [92], suggesting that CDC42 may be involved in regulating drug transport or receptor degradation, thereby regulating the endocytosis and efflux of chemotherapeutic agents in cancer cells.

Altered CP in cancer cells can also modulate the adaptive responses to chemotherapeutic agents, leading to the chemoresistance. For example, the LLGL2, which is overexpressed in ER^+^ BC cells, regulates levels of the cell surface leucine transporter SLC7A5 (solute carrier family 7 member 5) by forming a hetero-trimeric complex to promote the cellular uptake of leucine and cell proliferation. In contrast, ERs transcriptionally regulate LLGL2 expression, and BC resistance to endocrine therapy is associated with SLC7A5- and LLGL2-dependent adaptive responses to nutritional stress [93].

## CP-guided lymphocyte migration and differentiation

Increasing evidence suggests that CP proteins are actively involved in the maturation, migration, and functional activation of all lymphocytes, especially the immune effector cells, including T cells, NK, and macrophages. However, morphologically and biologically different from epithelial cells, T cells seem to share a similar mechanism regulating CP [94]. The regulation of T cell migration by CP proteins is initiated by chemokine receptor stimulation, which triggers adhesion molecule activation, such as LFA-1 (lymphocyte function-associated antigen 1) integrin, and orchestrates actin and microtubule cytoskeleton remodeling [95, 96]. Polarity regulators localized at the leading edge recruit signaling molecules like Rac1 and Cdc42 to establish cytoskeletal asymmetry and form a leading lamellipodium [97]. At the rear, the uropod concentrates ERM (ezrin, moesin and radixin) proteins that link membrane components with the cortical actin cytoskeleton and adhesion molecules like CD44 and ICAMs (intercellular adhesion molecules) interacting with integrins, including LFA-1 [98, 99]. In addition to regulating migration, CP proteins play a key role in asymmetric cell division (ACD), which allows the generation of both stem cells and self-renewing daughter cells [100]. It is applied in T cell ACD, in which the mother cell must first be polarized before division to generate two intrinsically different daughter cells. The ACD of T cells, recently well-reviewed by Kaminskiy et al., requires the coordinative function of a specific group of CP proteins that can guide T-cell morphological migration and differentiation [101, 102].

## CP proteins in immune cell tumor infiltration

Immune responses in TME are drawing increasing attention since checkpoint-based immunotherapy has remarkably changed the treatment strategies for various cancers. However, it is unknown if DCP is involved in exposing immune checkpoint expression that may affect cancer response to ICB. Regarding the large portion of unresponsiveness of solid tumors, including BC, it is important and highly informative to reveal the role of CP dynamics in influencing immune cell migration and tumor infiltration, as well as immune checkpoint protein richness and response to ICB.

**Fig. 3:**
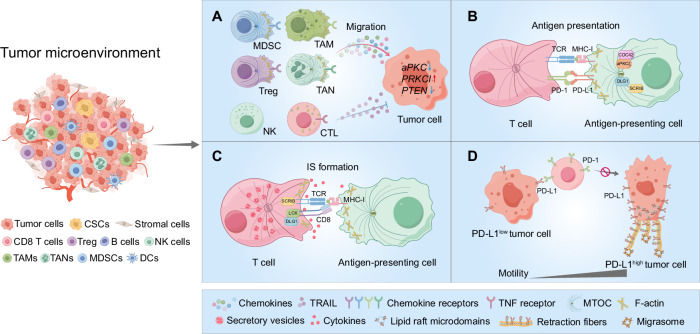
CP-associated immunosuppressive TME. TME consists of tumor cells, stromal cells, and infiltrating immune cells, including CD8 T cells, Tregs, B cells, NK cells, MDSCs, TAMs, TANs, and DCs. **A** Tumor cells recruit immunosuppressive cells (MDSCs, Tregs, TAMs, and TANs) and dysregulate CP, impairing the migration of cytotoxic immune cells. In tumors with disrupted CP signaling, such as aPKC-deficient intestinal cancers, PRKCI-overexpressing ovarian cancers, and PTEN-null glioblastomas, immune evasion occurs through MDSC infiltration and immune suppression, contributing to resistance to immunotherapy and supporting tumor progression. Additionally, tumor-derived TRAIL impairs T cell motility by lowering intracellular calcium, disrupting actin polymerization, and weakening adhesion to the extracellular matrix. **B** CP proteins (SCRIB, DLG1) regulate APC polarization and antigen presentation, with CDC42 and aPKCζ facilitating the formation of a microtubule-organizing center. **C** SCRIB and DLG1 transiently localize at the immunological synapse (IS), promoting T cell activation by clustering CD3 and PKCθ, and stabilizing TCR signaling through LCK interactions. **D** Tumor cell migration correlates with PD-L1 expression. In PD-L1-high tumor cells, PD-L1 accumulates at the rear, interacting with β4 integrin to form retraction fibers and avoid antibody targeting, reducing immune checkpoint blockade (ICB) efficacy. PD-L1-low tumor cells, PD-L1 are more evenly distributed, enhancing PD-1 interaction on T cells and improving ICB response. CP dysregulation in tumor and immune cells shapes an immunosuppressive TME and limits ICB effectiveness.

TME consists of cancer cells, including CSCs, along with a diverse array of non-tumor cells such as stromal cells, and a variety of infiltrating immune cells, including CD8 + T cells, regulatory T cells (Tregs), B cells, natural killer (NK) cells, myeloid-derived suppressor cells (MDSCs), tumor-associated macrophages (TAMs), tumor-associated neutrophils (TANs), and dendritic cells (DCs) (Fig. 3). Targeted migration of immune effector cells to the TME is a critical step of anticancer immunotherapy [103, 104], and aberrant immune cell migration facilitates tumor immune evasion and diminishes the effectiveness of ICB [104, 105]. Notably, alterations in polarity proteins have been implicated in shaping an immunosuppressive TME. For instance, the concurrent inactivation of both aPKCs in the mouse intestinal epithelium induces the spontaneous formation of aggressive microsatellite-stable (MSS) mesenchymal intestinal tumors. These tumors exhibit serrated and signet-ring carcinoma features, accompanied by reactive desmoplastic and immunosuppressive TME [106–108]. Furthermore, aPKC-deficient tumors demonstrate exclusion of CD8 + T cells to the stromal periphery, while being infiltrated by PD-L1 (programmed death-ligand 1)-expressing MDSCs, leading to resistance against anti-PD-L1 therapy [106]. However, in highly lethal serous ovarian cancers, overexpression of PRKCI drives immune evasion by inducing Yap1-dependent TNFα expression, which promotes MDSC infiltration and significantly suppresses cytotoxic T-cell activity [109]. Similarly, PTEN deficiency in glioblastoma cells activates Yap1, which upregulates Lysyl Oxidase (LOX) expression, attracting macrophages to the tumor microenvironment via the β1 integrin-PYK2 (protein tyrosine kinase 2-beta) pathway. This macrophage infiltration promotes glioma survival and angiogenesis, with LOX inhibition suppressing tumor progression and macrophage infiltration in PTEN-null glioblastoma multiforme (GBM) models [110] (Fig. 3A).

While alterations in tumor cell polarity proteins have been shown to influence immune cell infiltration, the role of polarity proteins in immune cells themselves during this process remains poorly understood. Through the manipulation of chemokine signaling and matrix-remodeling mechanisms, tumors can alter chemotactic and adhesive pathways. For example, TRAIL (TNF-related apoptosis-inducing ligand) reduces the motility of Jurkat cells (a T cell line) by lowering intracellular calcium levels, leading to actin filament depolymerization. Furthermore, TRAIL exposure diminishes the adhesion of Jurkat cells to the extracellular matrix protein laminin, thereby further impairing cell migration within laminin-rich environments [111] (Fig. 3A). It remains unclear whether and how CP functions in regulating the different activities of immune cells, specifically in the course of immune tumor infiltration. On the other hand, the dynamics of CP of tumor cells may disrupt T cell surveillance and the IS formation to tolerate the cytotoxicity of effector cells. With the emerging evidence of IS highly related to tumor immunosuppression and ICB efficacy, we discussed CP-associated IS formation in the following section. Upon antigen exposure by DC, aPKC and PAR3 polarize distally, while SCRIB and DLG localize proximally, facilitating T cell ACD. SCRIB, in particular, is vital for T cell CP and down-regulation of SCRIB-impaired T cell migration and antigen presentation [112], again underscoring the importance of CP and IS of polarity in T cell function.

## CP in tumor antigen presentation

SCRIB and DLG1, two major members of the PDZ protein family, play critical roles in the polarization of almost all epithelial cells. The functions of these two proteins are primarily investigated in T and B cells, in addition to studies of their expression patterns during antigen-presenting cell differentiation and DC maturation. Both proteins are required for conducting DC cell functions and are targeted by the influenza A viral protein NS1 in a PDZ-dependent manner, suggesting a potential role in innate immunity and antigen processing and presentation that can be exploited by viral pathogens [113, 114]. Furthermore, CP has been found in B lymphocytes where they play some key regulatory roles in antigen extraction, processing, and presentation [115]. Key components of the conserved polarity machinery, such as cell division CDC42 and aPKC zeta-type (aPKCζ), are essential for B cell polarization [116]. Disruption of either CDC42 or aPKCζ impairs the relocation of the microtubule-organizing center (MTOC) and lysosomes at the synaptic interface, leading to significant reductions in antigen extraction, processing, and presentation (Fig. 3B) [117]. An effective antitumor immune response depends on regulating CD4 and CD8 T cell activities mediated by antigen-presenting cells (APC) [118]. However, tumors often establish an immunosuppressive microenvironment that shields them from immune recognition. This immunosuppressive effect frequently stems from the inability of APCs, particularly dendritic cells, to recognize, process, and present tumor antigens to T cells [119, 120]. While there is currently no direct evidence linking CP components to the formation of immunosuppressive microenvironments, their roles in maintaining APC function are of significant interest (Fig. 3B). Proteins such as SCRIB and DLG1 are crucial for the proper polarization of APCs, which is essential for efficient antigen capture, processing, and presentation. Investigating how these CP proteins influence APC functionality in tumor contexts could provide insights into enhancing T-cell activation and improving the efficacy of ICB (Fig. 3B).

In addition to its roles in APCs, CP may also influence antigen presentation in tumor cells, potentially allowing tumors to evade immune surveillance. A critical mechanism by which tumors achieve immune evasion involves the suboptimal expression of major histocompatibility complex (MHC) molecules and components of the antigen processing machinery (APM), such as LMP2 (large multifunctional peptidase 2), LMP7 (large multifunctional peptidase 7), TAP (transporter associated with antigen processing), and tapasin [121–123]. While there is substantial evidence supporting the role of these components in tumor immune evasion [124, 125], the specific impact of CP disruption on the localization and function of these antigen-presenting components in tumor cells remains an area requiring further investigation. It is worth exploring whether CP components like SCRIB and DLG1 affect the localization and function of major histocompatibility complex (MHC) molecules and other APMs in tumor cells.

## CP in immunological synapse formation

Increasing evidence indicates that the efficacy of ICB is tightly associated with IS that is formed by the availability and affinity of related receptors of tumor cells or APCs with cytotoxic effectors [126–128]. Although the CP has not been demonstrated with tumor immunosuppressive function nor resistance to ICB, the dynamics of CP regulated by CP protein relocation in T cells could play a critical role in such IS formation. Key CP regulators are integral to T cell IS formation, underscoring the potential impact of CP on immune responses against tumors. For instance, the polarity proteins SCRIB and DLG1 transiently accumulate at the IS shortly after their formation, only to be excluded later. Knockdown of SCRIB disrupts the clustering of CD3 and PKCθ (protein kinase C theta) to anti-CD3 + CD28 stimulatory beads, crucial for T cell activation [112]. DLG1 supports T cell activation by interacting with the TCR (T-cell receptor) signaling complex via the kinase Lck, facilitating actin polymerization, NFAT (nuclear factor of activated T-cells) activation, and cytokine production through the p38 MAPK pathway [129, 130] (Fig. 3C). These functions of CP regulators like SCRIB and DLG1 highlight their importance in IS formation and, by extension, in the immune response against tumors. It is plausible that tumors might exploit these CP-related pathways to modulate immune responses, potentially leading to impaired IS formation.

Another crucial aspect of CP regulation is the precise control of vesicle trafficking between the cytoplasm and plasma membrane, which is vital for maintaining polarity and function in both motile and activated T cells. In CTLs, secretory vesicles containing cytotoxicity granules are oriented towards the TCR at the IS [131], while vesicles carrying cytokines such as IFN-γ (interferon-γ) and IL-2 are directed towards the APC [132] (Fig. 3C). Proper alignment of CP in cytotoxic T lymphocytes (CTLs) is essential for directing lytic granules towards APCs or tumor cells, a process critical for effective tumor cell killing. A recent work by Li et al. indicates that Her2-expressing brain metastasis can escape the cytotoxicity attacks by NK and CD8^+^ T cells via acetylation of lamin A-K542 mediated by nervous system-enriched metabolite N-acetyl aspartate (NAA) leading to reduction of the polarization of lytic granules and IS formation, compromising ICB combined with anti-Her2 therapy [133]. This finding underscores the importance of CP dynamics in IS formation and reveals a potential mechanism by which tumors might overcome immune surveillance and therapeutic interventions.

## CP proteins as therapeutic targets for reversing TME immunosuppression

Specific CP patterns may enhance the immune checkpoint protein expression and interaction to defend tumor cells from immune surveillance. Accumulating evidence suggests that the migration dynamics of normal cells are shared by cancer cells for migration, invasion, and tumor metastasis. FRP formation for normal cell migration is induced by relocated CDC42 which recruits PAR6, aPKC, and adenomatous polyposis coli to the microtubule tail, and PAR3 which activates related RAC1 [134–136]. Cell migration is also accelerated by PATJ front-localization [137], and the CRB family proteins which regulate the cytoskeletal actin backbone [138]. The front-localized SCRIB promotes actin polymerization and the formation of cell protrusions in migratory cells [139], where DLG1 interacts with adenomatous polyposis coli in a SCRIB–or PAR complex-dependent manner to stabilize microtubule ends [135]. n ER^-^/PR^-^ BC, aPKC is linked with metastasis [140], and aPKC-promoted TNBC metastasis is found via inhibiting E-cadherin and the TJ factor ZO1 (zonula occludens 1) [141, 142]. Blocking aPKC inhibits spontaneous migration and metastasis [143]. Interestingly, the N-terminus of SCRIB is activated in aggressive BC, with the proline-rich C-terminus lost [144], and SCRIB is favorably localized to the cellular protrusions in metastatic cells; blocking SCRIB inhibits the FRP and tumor metastasis [10]. Targeting anti-tumor CP protein CRB3 can inhibit tumor growth and lung metastasis [43], and LKB1 overexpression suppresses growth, microvascular density, and lung metastasis in LKB1-deficient MDA-MB-435 cells [145]. However, targeting CP proteins poses a challenge due to potential side effects on normal cells due to shared CP mechanisms. Importantly, the mislocalization and dysregulation of CP proteins impact asymmetric and symmetric cell division (SCD) in CSCs, which influences tumor heterogeneity and resistance to therapies. However, a dilemma in targeting CP proteins is related to the potential side effects on normal cells due to the shared CP proteins and mechanisms. Nevertheless, targeting CP for the control of circulating tumor cells (CTCs) could be doable based on the report that enhanced single-cell polarity detected in the CTCs is associated with metastatic potential [146].

## CP in tumor immunosuppression and resistance to ICB

ICB and ICB combined therapeutic modalities are increasingly applied in clinical cancer control, including BC treatment [147, 148]. CP may play a crucial role in tumor response to ICB by affecting the accessibility of immune checkpoint receptors. Contrasted with the extensively studied polarization of immune cells in physiological and pathological conditions [14, 149–151], it is currently unknown how CP proteins are involved in the exposure of either immune checkpoint receptors or antigen presentation. Affected interaction between tumor and immune cells due to alternations of CP in both or either side may play a key role in cancer response to the ICB or ICB combined anti-cancer therapies. Tumor CP dynamics, which drive cell migration and metastasis, may also affect the availability and richness of immune checkpoint proteins on the cell membrane, potentially limiting the recognition and affinity with the active immune cells. Interestingly, a recent study showed that the classical immune checkpoint protein PD-L1 is concentrated at the rear of migratory cancer cells, where it interacts with β4 integrin to stimulate contractility and form PD-L1-containing retraction fibers and microsomes [150, 152] (Fig. 3D). Notably, CP proteins SCRIB and DLG1 are demonstrated to regulate the antigen presentation in DC, indicating that CP of cancer cells can modulate the polarized localization of tumor antigens to avoid recognition by DC or macrophages. The EMT transition of tumor cells, as stated to be mediated by altered CP, could also lead to a suppressive TME characterized by an enrichment of suppressive immune cells, checkpoint proteins, and immunomodulatory chemokines [149]. These aspects explain how cancer cells escape from immune attack and may cooperatively contribute to the TME response to ICB therapy. Based on these assumptions, we raise a prospect here that targeting CP-associated proteins in combination with ICB may enhance IS formation and synergize BC response to ICB. To achieve this, a much broader and more comprehensive mode of action is to be established and validated, as it is still unclear whether and how the functions of CP proteins in cell-cell communication are linked to immune checkpoint upregulation or chemokine production in the TME. Moreover, CP dynamics influence the localization of immune checkpoint proteins, such as PD-L1, on tumor cells. For example, PD-L1 localization at the rear of migratory cancer cells interacts with β4 integrin, forming retraction fibers and migrasomes, thus limiting immune recognition. Such alterations in CP not only impact tumor progression but also modulate the immune microenvironment, highlighting the interconnected roles of CP in breast cancer and immune function. These insights indicate a potential critical mechanism by which cancer immunosuppressive status may be directly associated with the cellular polarities of infiltrated immune cells. Further exploration on topics such as whether the infiltrated immune cells in the TME could be affected by the host tumor cells with the relocation of the CP proteins or deficient or mutant CP genes is expected.

## Conclusion

**Fig. 4:**
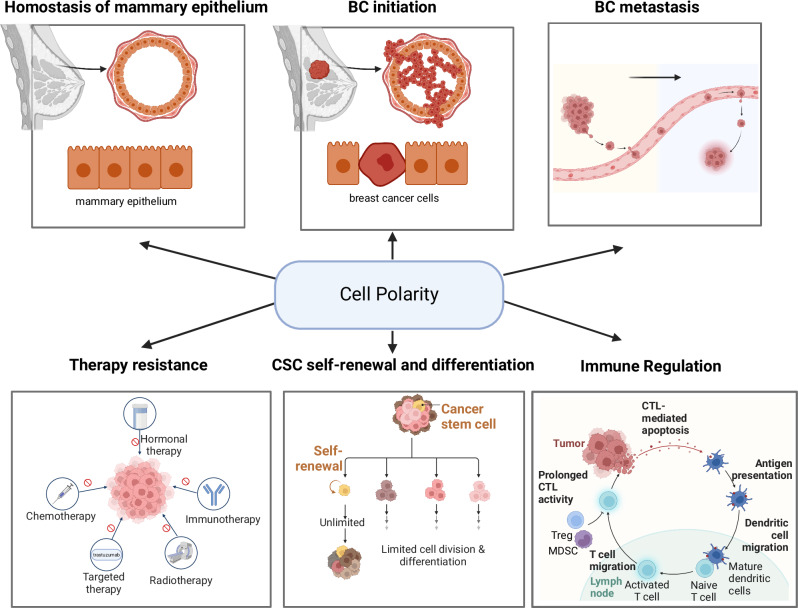
CP-associated BC initiation, progression, and immunosuppression. This review discusses a putative unique mechanism of CP-regulated immunosuppressive tumor microenvironment in breast cancer. By revisiting the model of CP-mediated mammary epithelial cell transformation and progression and CP-driving immune cell motivation and migration, we highlighted the multiple aspects of CP proteins in mammary gland luminal homeostasis and CP-associated BC initiation, progression, and metastasis. Emerging evidence is addressed in CP-mediated cancer stem cell repopulation and tumor resistance. A potential critical mechanism of CP-mediated immunosuppressive TME is discussed based on the CP-mediated disorganization of checkpoint receptors limiting the interaction/recognition between ACPs and active immune cells and critically the formation of IS, a key step in antigen presentation and tumor attacks by cytotoxic immune effector cells. Such CP-induced cellular polarity in tumor cells and immune cells, especially the number and kind of TIL, may play an essential role in breast cancer immunosuppression, leading to resistance to ICB.

This review integrates established and emerging insights into the role of CP proteins in mammary epithelial homeostasis and its potential critical functions in the interaction between tumor and immune cells immune cell function (Fig. 4). CP proteins are pivotal in maintaining luminal homeostasis. Their dysregulation can lead to significant disruptions in epithelial structure, contributing to malignant transformation and aggressive tumor behavior. Aberrant localization and interactions of CP proteins with various signaling pathways can compromise epithelial integrity, thereby driving tumor progression and enhancing the invasiveness of BC cells via boosting cancer stem cells and BC metastasis. Notably, this review extends CP dysregulation beyond tumor progression to immune cell dynamics within the TME. Specifically, alterations in CP proteins affect immune cell migration and infiltration, potentially fostering an immunosuppressive TME.

Furthermore, CP-associated changes in neoantigen expression on BC cells and modifications in antigen presentation by immune cells are notable. These disruptions in antigen presentation may facilitate immune evasion and diminish the effectiveness of ICB therapies. Finally, the role of CP proteins in forming IS underscores their impact on immune cell activation and function. Addressing these challenges, CP-targeted therapies present a promising yet complex frontier. Due to their critical roles in maintaining cellular architecture and regulating signaling pathways, CP proteins are potential therapeutic targets. However, issues such as specificity, systemic effects, and the heterogeneous nature of BC complicate their use. Integrating CP-targeted therapies with ICB could enhance therapeutic efficacy by boosting immune responses against the tumor, but this approach requires careful optimization to balance efficacy and minimize adverse effects. Further revealing the dynamics of IS formation regulated by CP on both tumor and immune cells may invent new effective strategies for BC immunotherapy. Future research should focus on elucidating these mechanisms to better harness CP-targeted therapies in clinical settings.

## Data Availability

All data generated or analyzed during this study are included in this published article.
